# Weldability Assessment of Various Steels by Hard-Facing

**DOI:** 10.3390/ma15093082

**Published:** 2022-04-24

**Authors:** Dušan Arsić, Vukić Lazić, Ružica R. Nikolić, Norbert Sczygiol, Božidar Krstić, Djordje Ivković, Branislav Hadzima, Filip Pastorek, Robert Ulewicz

**Affiliations:** 1 Faculty of Engineering, University of Kragujevac, Sestre Janjić 6, 34000 Kragujevac, Serbia; dusan.arsic@fink.rs (D.A.); vlazic@kg.ac.rs (V.L.); bkrstic@kg.ac.rs (B.K.); ivkovic99djordje@gmail.com (D.I.); 2 Research Centre, University of Žilina, Univerzitna 8215/1, 010 26 Žilina, Slovakia; branislav.hadzima@uniza.sk (B.H.); filip.pastorek@uniza.sk (F.P.); 3 Faculty of Mechanical Engineering and Computer Science, Czestochowa University of Technology, 42201 Czestochowa, Poland; norbert.sczygiol@pcz.pl; 4 Department of Production Engineering and Safety, Czestochowa University of Technology, 42201 Czestochowa, Poland; robert.ulewicz@pcz.pl

**Keywords:** weldability, carbon steel, alloyed steel, hardness, microstructure, preheating

## Abstract

Two aspects of various steels’ weldability are considered in this article. The theoretical part presents general concepts related to steel’s weldability and the application of the most important methods for its determination. In the experimental section, results of the hard-facing application to several samples are presented, and consist of hardness measurements in the different zones of the welded samples, with the evaluation of those zones’ microstructures. The tested materials included two carbon steels and two alloyed steels, with hard-facing layers deposited by various filler metals. Experimental results were compared to results obtained by calculations; using both, authors were able to conclude which combination of filler metal, welding procedure and, if necessary, heat treatment, would achieve the optimal improvement of weldability in welding/hard-facing of each of the tested base metals.

## 1. Introduction

Material weldability is an area of material science in which research into the design and execution of the welding and accompanying technologies is very important. During welding, very complex phenomena occur in the material; the knowledge of which is necessary for successfully welding. Owing to existence of numerous welding methods, it is now possible to successfully join various materials into an unbreakable whole.

Weldability is a complex property of metals, which has led to the emergence of several different definitions of the term. However, a unique and explicit definition of weldability, which would be extremely important for the practice of this phenomenon, does not exist. The weldability determines the relative capability of a certain material to form joints of the required quality level by the application of the corresponding procedure and technology. During welding, certain materials can possess good weldability, conditional weldability, or poor weldability.

The estimation of the weldability of various metals is of a great importance, both during the selection of filler metals (FM) and methods for manufacturing the welded structures, as well as during the selection of filler metals and procedures for reparation of damaged workpieces of various constructions. Estimating weldability based on a single method is not reliable. The contemporary approach to weldability estimation includes the following: the application of the computer methods that are taking the limit conditions into account, tests with simulations of the temperature cycles during the welding/hard-facing, and tests on samples with the real welding parameters. Determination of the chemical composition, hardness, and toughness of the base metal (BM), as well as of the filler materials and the welded joint as a whole, constitute preparation for the welding technology definition. For determination of the optimal welding technology, one needs to perform checking tests of the hard-faced workpieces in exploitation conditions.

Weldability can be estimated in various ways; the selected procedure most frequently depends on the type of welded material. If the subject of investigation is carbon steel, in practice it often suffices to perform a calculation check for weldability, which assumes application of various expressions based on the carbon equivalent (CE). However, for the steels of a more complex chemical composition (alloyed steels, high-strength steels, etc.), methods of obtaining the weldability estimates usually also require experimental investigation [1,2,3,4].

The experimental results of the weldability estimates for the three types of steels–structural, medium-carbon, and low-alloyed–are presented in this paper, after the presentation of the theoretical calculations. A comparison of the two approaches is presented, as well as the verification of the former.

## 2. Literature Review

Many researchers have considered problems related to weldability from various aspects. Those include estimating weldability value from different formulas for a particular material, evaluating the influences of different mechanical and physical properties, both of the base metals and the filler materials, on improving/worsening weldability, as well as the influence of different welding procedures and heat treatment (preheating or post-welding treatment) and the addition of different alloying elements to steels. This review is limited to references related to the problem considered in this paper; for the sake of brevity, this information is given in the form of a table describing the problems that the researchers were considering and results that they reported (Table 1).

## 3. Theoretical Weldability Estimates and Preheating Temperature Calculations

### 3.1. Base Metals

The following four steels were used for experimental research: S235JR, S355J2G3, C45, and 42CrMo4 (according to standard EN 10025-1 [26]). The first two steels, S235JR and S355J2G3, belong to a group of general structural specially tempered steels. They are used for production of the so-called responsible parts of welded structures, forgings and other highly stressed parts in mechanical engineering (axles, shafts, spindles, gears, and worm gears, etc.). Those steels are also used for the manufacture of various constructions: bumper-fences, girders, etc. C45 steel belongs to a group of carbon steels for tempering and has a wide applicability in the production of various parts of technical systems, in the construction machinery and for snowplows, knives for graders and bulldozers, parts of the loading machines, parts for spreaders and sprinklers, as well as for screws, nuts, etc. The steel is delivered in various shapes, such as rods of different cross-sections and sheets of medium and larger thicknesses The 42CrMo4 steel belongs to a group of the low-alloy tempered steels and it is most often used for production of the highly loaded parts of technical systems, such as vehicle and machine semi-axles, engine cylinders, cardan gears, etc. In addition, it is also used in construction mechanization, including for production of the lawn mower blades and blades for plant cleaning devices, gears, rotating toothed rims, toothed rails, etc. It is very important to have complete knowledge of the material that is to be repaired so that the reparation results are optimal [27]. Table 2 gives a summary of the chemical composition of the four analyzed steels, while Table 3 shows their mechanical properties and microstructure [1] as prescribed by the manufacturers.

### 3.2. Weldability Estimates and Preheating Temperature Calculations

The notion of weldability describes a complex property, simultaneously related to material, technology, and construction, which governs the execution of a welded joint with the required properties. Which of the properties are required depends on the purpose of the welded joint; in a certain case that may be uniformity of the mechanical properties; in the second case, the uniformity of the corrosion properties; while in the third, the uniformity of electrical properties, etc. Considering the complexity of the notion of weldability, one distinguishes between the metallurgical weldability, which relates to material being suitable for welding, the technological weldability–related to material being able to be welded by a certain procedure and the construction weldability that refers to reliability of the welded joint, welded assembly or the welded structure as a whole. Increased content of carbon and alloying elements worsens the steel’s weldability. If the carbon content surpasses 0.25%, a material is considered as conditionally weldable. The weldability is additionally worsened by the alloying elements, which in turn are increasing some other properties of steel.

Several methods exist for weldability estimates of various types of steels, which are all based on calculating the so-called equivalent carbon. For the low-alloyed steels, the content of carbon rarely exceeds 0.25%, while the alloying elements, which improve the useful properties of steels, worsening their weldability; the most commonly used formula are (1) given by International Institute for welding (IIW) and (2) by the American Welding Society (AWS) [8].

(1) $$CE=C+\frac{Mn}{6}+\frac{Cr+Mo+V}{5}+\frac{Ni+Cu}{15},\;\;\%$$

(2) $$CE=C+\frac{Mn+Si}{6}+\frac{Cr+Mo+V}{5}+\frac{Ni+Cu}{15},\;\;\%$$

Steels with high *CE* values can be hardened due to effect of the welding temperature cycles. If the hardness in a welded joint exceeds certain limit, which depends on the quantity of the diffused hydrogen, cracks will appear. That is why the limiting values of hardness are related to the content of the diffused hydrogen (given in ml per 100 g of the weld metal): for H_2_ = 20, the hardness limit is 350 HV; for H_2_ = 10 to 20, the hardness limit is 375 HV; for H_2_ = 5 to 10, the hardness limit is 400 HV; for H_2_ = 1 to 5, the hardness limit is 450 HV [22,23,28].

By applying the expression obtained by the statistical processing of the experimental results: HV = 1200 × CE-200, one obtains the limiting value of the equivalent carbon of 0.45%. Thus, if *CE* < 0.45%, the steel possesses good weldability. On the other hand, if *CE* > 0.45%, that steel is conditionally weldable. This term means that additional heat treatment would be required to obtain a welding joint of the required quality. A more detailed classification of weldability, in terms of the *CE* value, is given in Table 4, together with recommendations on the necessity of applying preheating procedures.

Besides the content of alloying elements, the thickness of the workpiece to be welded affects the weldability, since it influences the cooling rate of the joint. For instance, for the same base metal heated up to 200 °C, for the workpiece of thickness of 12 mm, the cooling rate is 28 °C/s, while for the workpiece of thickness of 20 mm, the cooling rate is 5 °C/s). This is why the formula for the total equivalent carbon was introduced by Séférian [29]:

(3) $$[C]={\left[C\right]}_{h}+{\left[C\right]}_{s}={\left[C\right]}_{h}\cdot \left(1+0.005\cdot s\right)$$

where *s* (mm) is the material thickness and:

(4) $${\left[C\right]}_{h}=C+\frac{Mn+Cr}{9}+\frac{Ni}{18}+\frac{7\cdot Mo}{90},\;\;\%$$

or according to formula (5). Here, [*C*]*_s_* is the carbon equivalent depending on thickness, while [*C*]*_h_* is the carbon equivalent depending on the content of the diffused hydrogen.

For the conditionally weldable steels, preheating is recommended, where the preheating temperature *T_p_* is calculated according to Séférian [29]:

(5) $$T_{p}=350\cdot \sqrt{[C]-0.25},\;\;({}^{\circ}C)$$

Based on data for the base metals, given in Table 1, the calculated values of *CE* for the base metals were: for S235JR-0.18% according to formula (1); for S355J2G3-0.47% according to formula (1); for C45-0.53% according to formula (2) and for 42CrMo4-0.63%-according to formula (3). The thickness value used was 20 mm, which is the diameter of the hard-faced bar. Except for S235JR steel, the weldability of which was characterized as good, other steels were conditionally weldable and required additional heat treatment. The preheating temperatures, calculated according to Séférian’s formula, are given in Table 5.

## 4. Experimental Investigation on Samples

These experimental tests were conducted to obtain a clearer picture of carbon and alloy steels’ weldability. The main objective was to perform the successful repair or production welding of different types of steels, by applying the appropriate procedure, selecting adequate filler metals, and designing the optimal welding technology [28,30]. Experiments were performed on the samples obtained by welding bars of selected carbon and alloy steels. The experimental research included: welding of steel specimens, preparation of welded specimens for testing, and determination of hardness and assessment of microstructure. The properties of the four filler metals used in experimental research are presented in Table 6 and Table 7.

### 4.1. The Filler Metals

For the experimental hard-facing the filler metals used were produced by “SŽ-Elektrode Jesenice” [31], the chemical composition of which is presented in Table 5, while Table 6 contains the mechanical properties of the pure weld metal obtained by welding with these filler metals. Prior to preparing the samples, tests of welding with various additional materials on various workpieces were performed. By varying the welding parameters (current power, operating voltage, speed, filler metals’ diameters, thickness of workpieces, etc.), the technological welding parameters were obtained that produced welded layers of a satisfactory quality.

The EVB 50 electrode is a basic electrode used for welding of the non-alloyed and low-alloyed steels and steel sheets of strength up to 610 MPa, as well as for the welding of the fine-grained high-strength steels. The welds produced by this FM are tough, even at low temperatures, and resistant to appearance of cracks. The hydrogen content in the weld metal is lower than 5 mL/100 g of the weld metal. The electrode has excellent welding and technological properties and a stable arc. The slag resulting during the welding can be easily removed.

Electrode E DUR 600 is a basic electrode alloyed with chromium and suitable for depositing the hard layers on steel parts that require very high wear resistance. It is used for the welding of parts of various machines, such as crushers, excavators, plow coulters, pneumatic tools, as well as for scissors, knives, tools for pressing, punching, and forging, and other tools for working in cold and hot exploitation conditions. The welds produced by this FM are tough and resistant to impact loads. This electrode can be used to weld both steels and steel castings.

The VAC 60 wire is a shielded welding wire for the GMAW process. It is suitable for welding both the non-alloyed and low-alloyed steels of strength up to 530 (MPa). It is also used for welding boiler sheets, pipes, shipbuilding steels, micro-alloyed steels, and steel sheets.

The FILTUB DUR 16 wire is a basic medium-alloyed cored wire, suitable for depositing the hard layers on parts that are exposed to high-intensity wear processes. The welds obtained by this FM are free of pores and cracks, which makes them resistant to varying dynamic and impact loads. It is used for welding of the blades and coatings of mixers, teeth, and other parts of construction machinery and parts of crushers, such as conical inlets, jaws, impact beams, housings, etc.

### 4.2. Preparation and Hard-Facing of the Samples

The preparation/cutting of the base metals for samples was performed by cutting on the machine saw with intensive cooling to prevent eventual structural changes due to excessive heating. The preparation of the filler metals for making samples refers to drying of electrodes at a temperature of 350–400 °C for 2 h. All the samples were made in the same laboratory conditions and the welding was performed in a horizontal position. The preheating temperature was controlled by a digital thermometer. The basic criterion for assessing the quality of the hard-faced layers was that the weld was homogeneous without pores, inclusions, or cracks, both in the weld metal and in the base metal (heat-affected zone).

The selection of the optimal surfacing parameters was not easy, since a large number of tests with different hard-facing parameters were required. Regardless, it was necessary to determine the optimal welding parameters, since only those can produce hard-faced layers of the required quality. After the testing, the optimal parameters were adopted and applied in sample manufacturing. These samples were used for testing of the welded layers in laboratory conditions. The hard-facing parameters for the samples produced by the MMAW procedure and coated electrodes are given in Table 8, while the process parameters for the samples produced by the GMAW procedure are given in Table 9. The hard-facing was performed in two layers.

## 5. Results

### 5.1. Hardness Measurements

**Figure 1:**
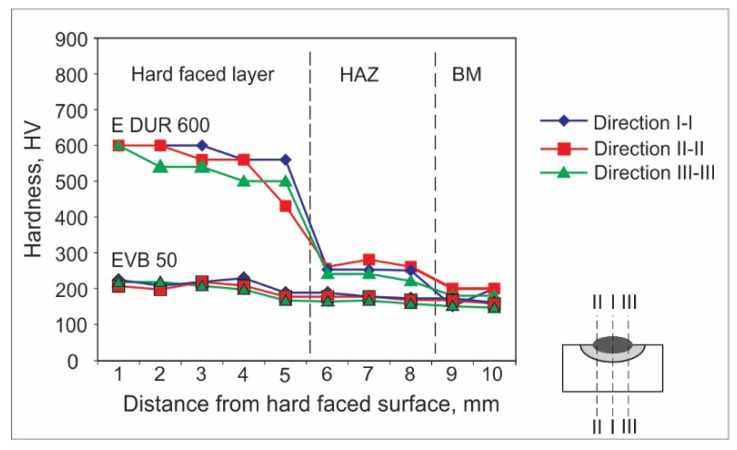
Hardness distribution in the hard-faced layer cross-section, BM-S235JR, FM-E DUR 600, EVB 50.

**Figure 2:**
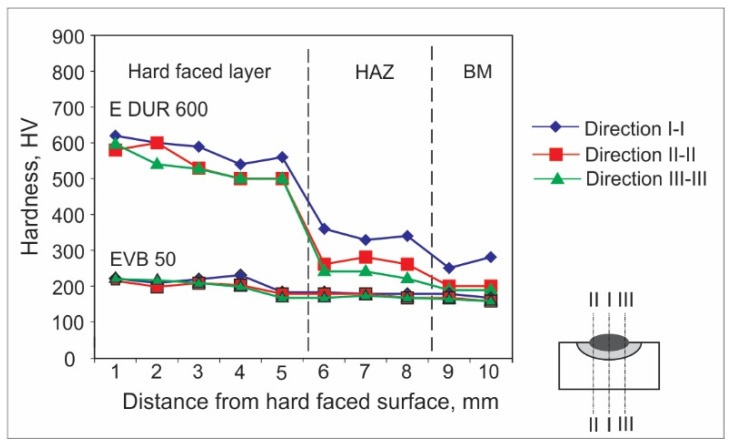
Hardness distribution in the hard-faced layer cross-section, BM-S355J2G3, FM-E DUR 600.

**Figure 3:**
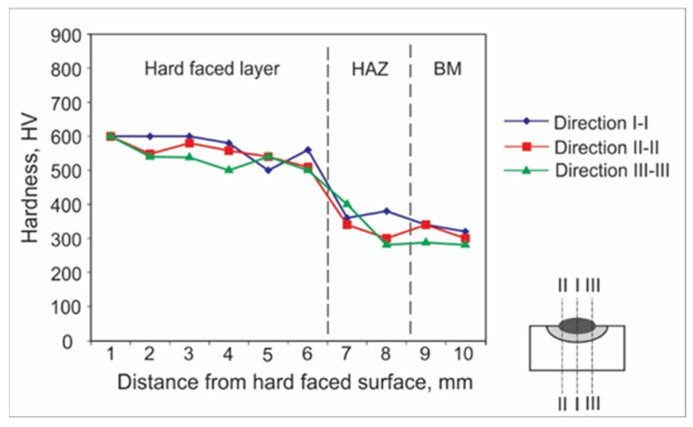
Hardness distribution in the hard-faced layer cross-section, BM-C45, FM-E DUR 600.

**Figure 4:**
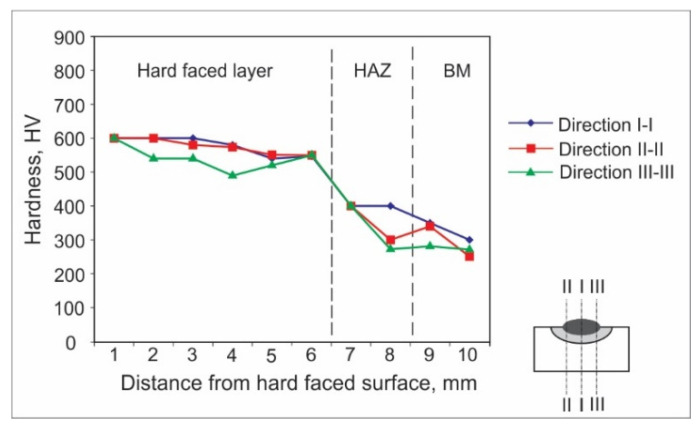
Hardness distribution in the hard-faced layer cross-section, BM-42CrMo4, FM-E DUR 600.

**Figure 5:**
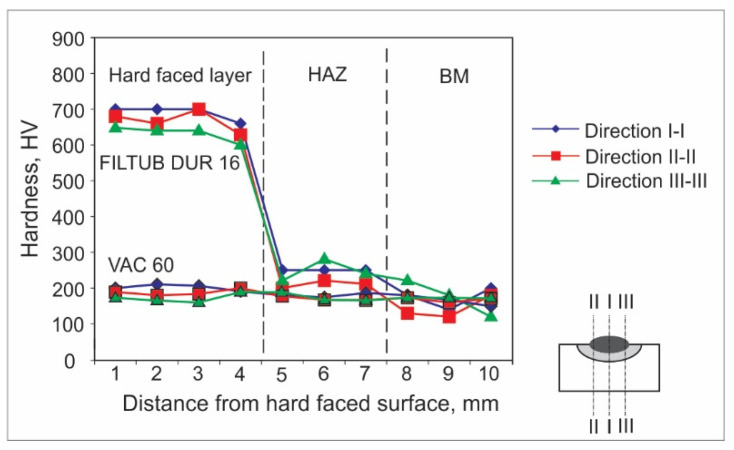
Hardness distribution in the hard-faced layer cross-section, BM-S235JR, FM-FILTUB DUR 16, VAC 60.

**Figure 6:**
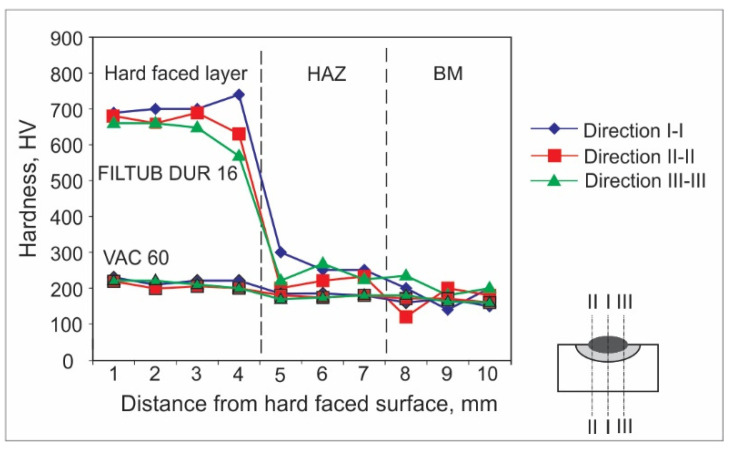
Hardness distribution in the hard-faced layer cross-section, BM-S355J2G3, FM-FILTUB DUR 16, VAC 60.

Hardness measurements can be achieved by various experimental methods. For welded joints and hard-faced layers, hardness is usually measured by the Vickers method due to a number of advantages; the main one is that this method has no restrictions on the value of the measured hardness. For the purpose of this research, the hardness measurements were performed in an accredited laboratory, using the Vickers method (pressing force F ≈ 100 N), and according to the appropriate standard. The obtained results are presented in the form of diagrams of hardness distribution along the hard-faced layer’s height in Figure 1, Figure 2, Figure 3, Figure 4, Figure 5 and Figure 6. The hardness of the weld’s characteristic zones is usually measured in three mutually parallel directions, marked as I-I, II-II, and III-III, passing through all the zones.

Figure 1 shows the hardness measurement results, obtained for hard-facing by the MMAW procedure, with electrodes E DUR 600 and EVB 50 of diameters d_e_ = 3.25 mm on the sample, the base metal of which was steel S235JR. The hard-facing was executed without using the preheating.

In Figure 1, it can be seen that the sample hard-faced by FM E DUR 600 possesses much higher hardness than the sample hard-faced with FM EVB 50. This is expected, considering the chemical composition of electrodes (Table 6). The high contents of carbon and chromium in the E DUR 600 electrode enable realization of the high hardness. The hardness of this sample is stable up to a depth of 5 mm, when the slight decrease in the base metal hardness occurs, whereas the hardness of EVB 60 is equal to the hardness of the base metal. This leads to the conclusion that the E DUR 600 electrode is suitable for hard-facing of parts that are in exploitation or subjected to wear; however, the thickness of the hard-faced layer, as well as intensity of its wear, must be monitored.

Figure 2 shows the hardness measurement results obtained for hard-facing by the MMAW procedure, with electrode E DUR 600 and EVB 50 of diameters d_e_ = 3.25 mm on the sample, the base metal of which was steel S355J2G3. The hard-facing was executed with preheating at 110 °C.

Figure 2 shows that the substrate (BM) has no influence on the surface hardness of the hard-faced layers, since the hardness of layers deposited by FM E DUR 600 is the same as for BM S235JR, the only difference being that hardness in the case of steel S355J2G3 is somewhat higher. Even the penetration depth (thickness of the hard-faced layers) is almost identical for both cases.

Figure 3 shows the hardness measurement results obtained for hard-facing by the MMAW procedure with preheating at 220 °C, with electrode E DUR 600 of diameter d_e_ = 3.25 mm on the sample, the base metal of which was steel C45.

When the case of the medium-carbon steel C45 is considered, from the analysis of a diagram in Figure 3, one can observe that the hard-facing penetration is 1 mm bigger than in the case of structural steels (Figure 1 and Figure 2) and the hardness of the HAZ and the BM is higher, which is to be expected due to the carbon amount. The hard-faced layer hardness is stable throughout its dept.

Figure 4 shows the hardness measurement results obtained for hard-facing by the MMAW procedure with preheating at 280 °C, with electrode E DUR 600 of diameter d_e_ = 3.25 (mm) on the sample, the base metal of which was steel 42CrMo4.

From the hard-facing of the low-alloyed tempered steel 42CrMo4 it was established that it behaves similarly to the C45 steel, with regard to both the hard-faced layer’s hardness and the layer’s thickness, as well as for the HAZ and BM hardness. It should be pointed out that hardness in the HAZ in both cases decreased gradually from 450 HV to 350 HV within the 2 mm thick layer, as shown in Figure 3 and Figure 4, while for the structural steels that is usually not the case, since their hardness drops abruptly (Figure 1 and Figure 2) and remains at the level reached.

Figure 5 shows the hardness measurement results obtained for hard-facing by the GMAW procedure without preheating, with cored wires FILTUB DUR 600 and VAC 60 of diameter d_e_ = 1.2 (mm) on the sample, the base metal of which was steel S235JR.

By analyzing the results shown in diagram in Figure 5, one can observe a big difference in hardness of the deposited hard-faced layers. From the comparison of results, there is the impression that the conclusion is the same as for the first case (Figure 1); the difference here is that the wire is used as the filler metal instead of the electrodes. The achieved hardness is around 700 HV, which is even somewhat higher than in the case of electrode E DUR 600; however, the hard-faced layer thickness is slightly smaller at around 4 mm. The VAC 60 wire is completely compatible with the used base metal. From the comparison of results it can be said that this method of hard-facing can be applied as a substitute for the electrode hard-facing.

Figure 6 shows the hardness measurement results obtained for hard-facing by the GMAW procedure with preheating at 110 °C, with cored wires FILTUB DUR 16 and VAC 60 of diameter d_e_ = 1.2 mm on the sample, the base metal of which was steel S355J2G3.

From the analysis of results presented in Figure 6, one can observe that for the case of GMAW hard-facing, the applied base metal has no influence on the output characteristics of the hard-faced layers; although there is a small difference only in the case of the BM hardness, everything else is quite similar.

### 5.2. Microstructure Analysis

**Figure 7:**
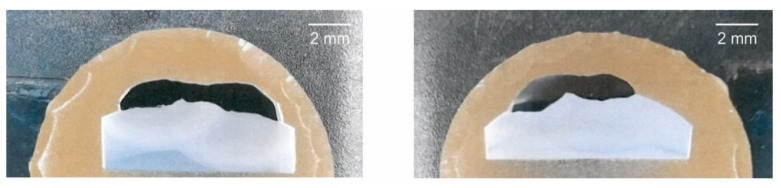
Appearance of some of the metallographic ground samples.

Within the conducted experiment, the bars made of those four steels (S235JR, S355J2G3, C45, and 42CrMo4) were hard-faced with two filler metals (E DUR 600 and FILTUB DUR 16). The metallographic cutoffs from the hard-faced bars are shown in Figure 7.

**Figure 8:**
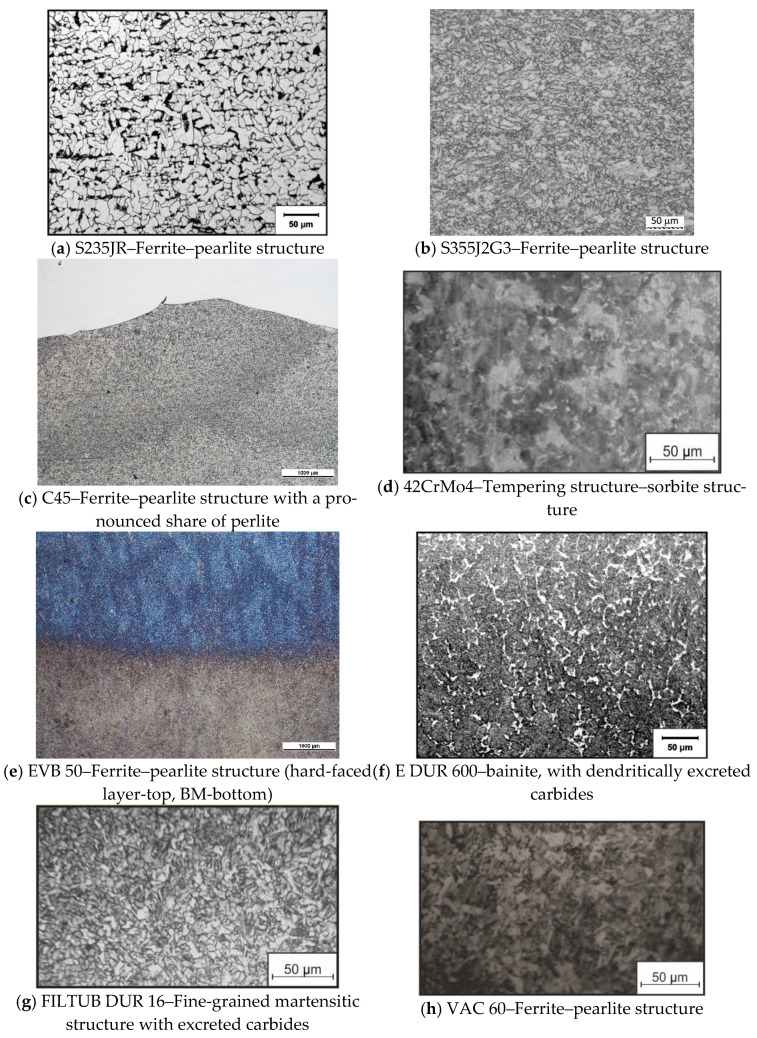
Microstructure in hard-faced layer characteristic zones: base metals (**a**–**d**); hard-faced layers (**e**–**h**).

Then, the microstructure of all the characteristic zones of the hard-faced layers was recorded on the metallographic microscope MCXM 500, with a magnification of 1000×. The results of each used BM and FM are presented in Figure 8.

The main structure present in the base metal was ferrite–pearlite, as shown in Figure 8a–c. In the case of the structural steels (Figure 8a,b), the presence of pearlite is somewhat smaller (due to lower carbon content in the steel), while for the steel for tempering C45 (Figure 8c), the presence of pearlite is more prominent. The 42CrMo4 steel (Figure 8d) is characterized by the tempering structure (sorbite).

When the subject matter is the filler metals (hard-faced layers), the EVB 50 electrode (Figure 8e) and electrode wire VAC 60 (Figure 8h) have the classical ferritic–pearlite structure owing to their chemical composition. For the case of the E DUR 600 electrode (Figure 8f), the situation is drastically different. Based on the optical metallographic analysis, it could be assumed that in the part of the surfaced layer of E DUR 600, immediately next to the melting line (in the dissolution zone), martensitic is the predominant microstructure, with dendritically excreted carbides. The structure is characterized as large-grain cast microstructure with primarily inhomogeneous dendrites of austenite. The dark color probably represents the (Fe,Mn)_3_C carbides excreted at grain boundaries. The similar situation is in the case of the electrode wire FILTUB DUR 16 (Figure 8g) where the structure is estimated as a fine-grain martensitic structure with excreted carbides. The structures of the filler metals FILTUB DUR 16 and E DUR 600 are as expected, considering their chemical composition (Table 6) and presence of the carbide-forming elements Cr and Mo. In addition, results of the measured hardness for these two cases (Figure 1, Figure 2, Figure 3, Figure 4, Figure 5 and Figure 6) completely correspond to the respective metallographic findings.

Through the metallographic investigations, it was established that there were no flaws in the hard-faced layers, such as cracks, inclusions, gas bubbles, and fusions of layers, which are a constant threat when performing hard-facing. To avoid such flaws, the whole set of measures was conducted, such as preheating, cleaning of the FM and BM prior to hard-facing, cleaning the individual layers after the hard-facing, etc.

## 6. Discussion

The experimental part was performed using samples in laboratory conditions. The hardness measurement of the individual hard-faced layer zones and the reading off of the microstructure were performed on samples prepared on a metallographic grinding wheel. Hard-facing was performed by depositing layers of the tested filler metals on various base metals. Electrodes, of trade names E DUR 600, EVB 50, as well as the cored wires FILTUB DUR 16, VAC 60, were used as the filler metals. The welding procedures used for hard-facing were MMAW (manual metal arc welding) and GMAW (gas metal arc welding).

For welding/hard-facing of samples made of the base metal steel S235JR, the depositing of layers was performed without the preheating, since this steel possesses good weldability. The carbon equivalent (CE) value for it was 0.18%, which is well below the limit of 0.45%. On the contrary, the other three tested base metals had carbon equivalent values above the limit value, which defines them as conditionally weldable. Thus, they needed additional heat treatment, which consisted of preheating at various temperatures, depending on their *CE* values. During welding of these steels, the products of hardening and high hardness, which are usually above the allowed limits, appeared in the HAZ. Through applying the preheating treatment, more favorable structures of lower hardness, higher toughness, and sufficiently high strength and yield stress, were obtained. That was accompanied by a reduction in the stress and strain levels in the material bulk. Due to the additional costs, the subsequent heat treatment should be avoided whenever possible.

Microstructural tests have shown favorable structures for welded specimens, free of cracks, porosity, adhesion, and other irregularities. The exception was the case of the welded steel sample 42CrMo4, where minimal porosity was registered in the interlayer. The appearance of porosity or inclusions between the welded layers may be a consequence of improper welding and deviations from the prescribed technology (welder’s error).

In addition, the experiment showed that hard-faced layers deposited with the filler metals E DUR 600 and FILTUB DUR 16 have higher hardness (about 600 HV) and are more suitable for welding than hard-faced layers deposited with filler metals EVB 50 and VAC 60, the hardness of which was approximately about 250 HV. That was expected considering the chemical composition of the filler metals and the established microstructure. Based on those results, it should be emphasized that the electrodes E DUR 600 and FILTUB DUR 16 can be used for hard-facing of parts exposed to intensive abrasive and adhesive wear, while application of other filler metals is not justified in such conditions, since their characteristics are close to those of the base metals. Those FMs can be used, for example in the case of a damaged workpiece, to replenish the missing material instead of replacing the whole workpiece.

## 7. Conclusions

Based on the theoretical and experimental research results presented in this paper, it was concluded that for the design of the optimal technology, complex and long-term research is needed due to various complex physical–chemical phenomena that occur during the melting and crystallization of alloys in the welding bath. These studies are necessary in order to obtain the required quality of the weld or hard-faced layers.

This experimental research has shown that the structural steel S235JR had good weldability and that no additional measures were required for its welding to obtain the required quality of the deposited layer. In contrast, steels S355J2G3, C45, and 42CrMo4 were conditionally weldable; thus, to improve their weldability additional measures are required, such as preheating before hard-facing and, in some cases, even a subsequent heat treatment (post heating).

The hard-faced layers obtained by the filler metals E DUR 600 and FILTUB DUR 16 had a hardness of 600 HV, which is significantly higher than 250 HV for the hard-faced layers obtained by the filler metals EVB 50 and VAC 60.

Microstructural tests have shown favorable structures in all the characteristic zones of the hard-faced specimens. No (cold or hot) cracks, porosity, adhesion, or other irregularities were recorded, except for the case of one sample of the 42CrMo4 steel, where minimal porosity was registered in the interlayer. In addition, the multi-layer deposition led to tempering of material in the previous layer, which led to a reduction in its hardness and to the formation of a more favorable structure. The last applied layer has the highest hardness, so if it is necessary, material from the surface part of the last deposited layer can be removed by grinding or some other machining, depending on its hardness.

Thus, the experimental results, through the values of the measured hardness of the hard-faced layers individual zones and recordings of their respective microstructures, confirmed the theoretical estimates on the weldability of individual steels, as well as confirmed that the preheating temperatures, for samples requiring heat treatment, were correctly evaluated.

The quality of a welded joint is affected by several different factors. After these experimental tests of the metallographic properties of welded layers, it can be concluded that the quality of the welded layer is mostly influenced by the welding technology applied. The adequate modeling of the welding/hard-facing process can create the conditions for obtaining hard-faced layers of the required quality.

## Figures and Tables

**Table 1:** Brief literature review.

No.	Article	Subject of Research	Main Conclusions/Contributions
[5]	Tolf and Hedegard (2007)	Possibility for improving the ultra HSS weldability in projection welding	By increasing the BM strength, the weldability and ductility of the joints become limited: the welding time vs the welding current balance is needed to avoid undersizing of the weld.
[6]	Talas (2010)	Assessment of carbon equivalent formulas	CE equations correlate highly with yield stress (YS), ultimate tensile strength (UTS), hardness (HRD), and elongation (EL%); thus, they are suitable for predicting mechanical and microstructural properties of steel weld metals.
[7]	Chang, Chen, and Wu (2010)	Microstructural and abrasive characteristics of the high carbon HF Fe-Cr-C alloys	Primary (Cr, Fe)_7_C_3_ carbide fraction increased with increased graphite addition, while their size decreased due to the increase in their nucleation rate.
[8]	Lin et al. (2010)	Influence of V, Mo, and Ni addition on the primary carbides’ morphology and mechanical properties and eutectic colonies in the Cr-Fe-C HF alloys	Adding vanadium, molybdenum, and nickel does not affect the morphologies of the primary carbides; however, their addition produces high-performance Cr-Fe-C hard-facing alloys.
[9]	Liu et al. (2013)	Influence of the boron content on the alloy’s microstructure and wear properties in HF of a mild steel	With an increase in boron content, the carbide average diameter increased from 9 to 20 (mm) and the carbide volume fraction (CVF) increased from 14.10 to 36.00%, causing an increase in the alloy’s hardness and abrasive wear resistance.
[10]	Tolf (2015)	Various parameters of the projection welding	The electrode force is an important parameter that must be correctly set to avoid excessive weld deformation.
[11]	Schipaanboord, Marquering, and Bruce (2015)	Application of low-yield filler metals for the safe welding of live gas pipelines	The parent material had to be buttered up with a low-yield electrode with at least two layers to avoid dilution with carbon, manganese, and silicon in the weld pool.
[2]	Arsić et al. (2016a)	Selecting the optimal HF technology based on the t_8/5_ cooling time	The weldability estimate can be reliably performed by use of continuous cooling transformation (CCT) diagrams based on use of the calculated t_8/5_ cooling time.
[3]	Lazić et al. (2016)	Weldability estimates for the C-Mn HSS	Optimal welding technology defined for HF of the tube girder cover made of the said steel.
[4]	Arsić et al. (2016b)	Testing of four filler metals under dry conditions	Evaluated tribological behavior of HF layers executed by different filler metals to define the optimal HF conditions.
[12]	Cabrilo and Geric (2016)	Weldability of the high hardness armor (HHA) Protac 500 steel	The optimal technology was defined by varying the welding procedures, filler metals, and heat treatment regimes.
[13]	Dobosy and Lukacs (2016)	Welding parameters effects on properties of the welded structures made of thermomechanically rolled HSS	All the welding parameters can be used within the wide range of values since their modification had a small effect on the properties of welded joints.
[14]	Han et al. (2019)	Weldability of dual-phase CMnSi steels in the resistance spot welding.	Due to formation of the internal (sub-surface) oxides during annealing, the surface oxide formation is suppressed and the resistance spot welding of the steel surface coated with zinc is affected.
[15]	Vicen, Bronček, and Novy (2019)	Possibilities of reducing the friction coefficient of bearing steel 100Cr6	Reducing the 100Cr6 bearing steel friction by coating with CarbonX DLC (diamond like) resulted in reduced wear and increased service time of the coated components.
[16]	Trško et al. (2020)	Weldability of the high-strength low-alloy (HSLA) steel Strenx^®^ 700 MC	The WM microstructure consisted of a fine acicular ferrite and the BM structure of a fine-grain rolled structure with Ti, Nb, and V carbides. The heat affected zone (HAZ) was less than 1 mm wide with significantly coarsened grains of polyhedral ferrite and carbides.
[17]	Krolicka et al. (2020)	Microstructure and wear behavior of claddings (Fe-Cr-C-Nb) on coulters, produced by commercial welding alloys	The claddings consisted of hypereutectic, near-eutectic, and hypoeutectic layers, with different primary M_7_C_3_ carbide content. The near-eutectic layer exhibited the most advantageous mechanical behavior.
[18]	Czuprynski (2020)	Abrasion resistance of the HF layer produced by the self-developed covered tubular electrode	Wear-resistance of 11 commercially produced plates were tested to obtain one with properties closest to those obtained by the new electrode.
[19]	Tomkow, Fydrych, and Rogalski (2020)	Various aspects of the wet-welding of the HSLA S460N steel	Effects of application of the waterproof coatings to electrodes on S460N steel’s weldability.
[20]	Tomkow and Fydrych (2020)		The hydrophobic coatings can reduce the hardness in the welded joints HAZ.
[21]	Tomkow (2021)		The temper bead welding (TBW) method can be applied for the wet-welding of this steel.
[1]	Ilić (2021)	Weldability of carbon and alloyed steels	To correctly obtain/evaluate weldability of a certain material, all the aspects must be taken into account.
[22]	Markovic et al. (2021a)	Influence of the FM type on performance of the regenerated cylindrical spur gears	The “hard” FM produces better characteristics for individual reparatory HF, while for the batch reparation of numerous damaged gears, “soft” FM hard-facing, followed by cementation and heat treatment, is more convenient.
[23]	Markovic et al. (2021b)	Influential phenomena during regeneration of parts to reverse their working ability loss	Filler metal types, the teeth geometrical accuracy, microstructure, and micro hardness were compared to properties of new gears’ teeth flanks.
[24]	Konat (2021)	Technological and structural aspects of welded joints of the Hardox 600 steel	The welding leads to formation of a wide HAZ, with structures favoring the reduction of abrasion resistance and deterioration of plastic properties, while increasing the susceptibility to brittle fracture. New effective welding technology is proposed.
[25]	Jilleh et al. (2021)	Microstructural development during solidification and the wear behavior of four hypereutectic white cast iron (WCI) HF deposits, on the carbon steel (SJ235RG2).	Addition of the MC carbide-forming alloying elements to the filler metal caused the grain refinement of the primary pro-eutectic M_7_C_3_ carbide, while the further grain refinement was caused by increased content of carbide formers (Nb, Mo). The deposits’ wear resistance increased with increased content of alloying elements in the filler metal.

**Table 2:** Chemical composition of the tested steels [1].

Base Metal	Chemical Composition, %								
	C	P	S	N	Si	Mn	Cu	Mo	Cr
S235JR	0.17	0.05	0.05	0.007	/	/	/	/	/
S355J2G3	0.23	0.035	0.035	/	0.6	1.7	0.6	/	/
C45	0.42–0.5	0.045	0.045	/	0.04	0.5–0.8	/	/	/
42CrMo4	0.38–0.45	0.035	0.0035	/	0.15–0.4	0.5–0.8	/	0.15–0.3	0.9–1.2

**Table 3:** Mechanical properties and microstructure of the tested steels [1].

Base Metal	Property						Microstructure
	R_m_ (MPa)	R_eh_ (MPa)	A5 %	Z %	KV (J)	Hardness (HB)	
S235JR	370–450	220–240	18–25	/	27	130–145	Ferrite–pearlite
S355J2G3	370–450	220–240	18–25	/	27	130–145	Ferrite–pearlite
C45	700–850	500	14	30	32	334–340	Tempered structure, predominantly tempered martensite *
42CrMo4	1100–1300	900	10	40	34	298–305	Tempered structure, fine pearlite with ferrite at grain boundaries

* Previously known as troostite.

**Table 4:** Weldability classes in terms of the *CE* value.

Carbon Equivalent (CE)	Weldability	Preheating
<0.35	Excellent	Not necessary
0.36–0.40	Very good	Recommended
0.41–0.45	Good	Necessary
0.46–0.50	Fair	Necessary
>0.50	Poor	Necessary

**Table 5:** The preheating temperatures of the tested steels.

Base Metal	Preheating Temperature, T_p_ (°C)	
	Calculated	Adopted
S355J2G3	~107	110
C45	~218	220
42CrMo4	~269	270

**Table 6:** Chemical composition of the tested filler metals.

Electrode Designation	Chemical Composition, %				
	C	Si	Mn	Cr	Mo
EVB 50	0.08	0.6	1.0	-	-
E DUR 600	0.5	-	-	7.5	-
FILTUB DUR 16	0.45	0.6	1.6	5.5	0.8
VAC 60	0.08	0.9	1.5	-	-

**Table 7:** Mechanical properties of the weld metal obtained by application of the tested filler metals.

Electrode Designation	Mechanical Properties of Pure Weld Metal				
	R_m_ (MPa)	R_eH_ (MPa)	A_5_, %	KV (J)	Hardness (HRC)
EVB 50	510–610	>440	>24	>47	-
E DUR 600	-	-	-	-	57–62
FILTUB DUR 16	-	-	-	-	57–62
VAC 60	510–590	>410	>22	>47	-

**Table 8:** Process parameters for the MMAW hard-facing.

Filler Metal Mark	Electrode Diameter d_e_ (mm)	Current, I (A)	Working Voltage U (V)	Speed, vz (mm/s)	Driving Energy, q_l_ (J/mm)
EVB 50	3.25	100–150	20–23	1.19–2.20	2016.8–2545.5
E DUR 600	3.25	100–150	20–23	1.19–2.20	2016.8–2545.5

**Table 9:** Process parameters for the GMAW hard-facing.

Filler Metal Mark	Protective Gas Flow (L/min)	Wire Diameter d_w_ (mm)	Current, I (A)	Working Voltage, U (V)	Speed, v_z_ (mm/s)	Driving Energy, q_l_ (J/mm)
EVB 50	16–20	1.2	130–150	23–28	35–80	3187.5–1333.0
E DUR 600	16–20	1.2	130–150	23–28	35–80	3187.5–1333.0
